# Dual SwinUNet architecture for enhanced photoacoustic imaging: a contrastive learning approach in image and sinogram domains

**DOI:** 10.1117/1.JBO.31.7.076002

**Published:** 2026-07-08

**Authors:** Isha Munjal, Tekeshwar Hirwani, Jaya Prakash

**Affiliations:** a Indian Institute of Science, Department of Instrumentation and Applied Physics, Bengaluru, Karnataka, India; b Indian Institute of Science, Department of Electrical Communication Engineering, Bengaluru, Karnataka, India

**Keywords:** contrastive learning, image reconstruction, photoacoustic imaging, Swin-UNet, transformers

## Abstract

**Significance:**

Leveraging complementary information from multiple data representations enables richer feature extraction and greater robustness under challenging acquisition conditions. Such an approach enhances the accuracy, reliability, and generalizability of photoacoustic image reconstruction, ultimately leading to improved image quality and stronger performance across diverse photoacoustic imaging (PAI) scenarios.

**Aim:**

We propose a transformer-based dual SwinUNet architecture that learns features from both the image and sinogram domains to improve PAI within a contrastive learning framework.

**Approach:**

The developed dual SwinUNet architecture had multiple loss function—wherein noise-to-signal ratio was computed between the predicted output from each SwinUNet model and the ground truth, and the mean square error was estimated between the predicted outputs of both networks. The dual SwinUNet model was fed with two reconstructed images as inputs, generated using different reconstruction algorithms, i.e., backprojection and Tikhonov-regularized reconstruction. These input pairs can either be positive, meaning both inputs share the same ground truth, or negative, meaning they have different ground truths. The model was trained using a contrastive loss in the sinogram domain, enabling the model to learn distinctive features from both positive and negative pairs.

**Results:**

The performance of the different networks (ResNet, UNet, FDUNet, TNet, and the proposed network) was evaluated by varying the number of transducers, angular coverage, and noise levels. The data acquired with 100 transducers having a coverage angle of 135 deg have shown that the structural similarity index measure (SSIM) was improved by 7.5% and the universal image quality index improved by 18% compared with FDUNet. For *in vivo* mice data, SSIM improved by 6.8%, when using data from 100 transducers.

**Conclusions:**

The dual SwinUNet architecture demonstrates significant improvement in image quality for PAI by learning features from both the image and sinogram domains. The proposed framework can be extended using different DL architectures alongside different analytical/model-based reconstruction inputs.

## 1 Introduction

Photoacoustic imaging (PAI) offers a unique combination of intrinsic molecular-level contrast of optical imaging and high (and scalable) spatial resolution of ultrasound imaging.^1^ PAI involves illuminating a biological tissue sample with a short nanosecond laser pulse, causing the tissue to absorb transient light energy and undergo thermoelastic expansion. This expansion generates acoustic waves, which are detected with an ultrasound transducer. The detected ultrasound signals can be used to localize the acoustic sources (also called the initial pressure rise) in the medium using an image reconstruction algorithm. The intensity of the acoustic source is related to the tissue’s optical absorption properties.^2^ Traditional PA image reconstruction methods often rely on algorithms such as filtered back projection (FBP),^3^^–^^5^ model-based methods,^6^^–^^8^ and iterative algebraic reconstruction techniques (ART).^9^^–^^11^ Although successful, FBP suffers in high-noise cases and limited-data scenarios, and iterative schemes tend to be computationally expensive (inhibiting real-time PAI).

FBP assumes regular data acquisition geometries (e.g., planar and spherical) and introduces artifacts when dealing with complex geometries.^4^^,^^12^^,^^13^ ART is better suited for complex geometries and tends to be more robust against noise but is computationally expensive (particularly for three-dimensional PAI). Although these techniques have been successful in PA image reconstruction, they may fall short in terms of accuracy or efficiency when dealing with complex geometries, noisy, or limited data. In recent years, deep-learning-based approaches have emerged as a promising alternative for PA image reconstruction, offering faster and more accurate results.^14^^–^^16^ These methods can learn intricate relationships between the measured ultrasound data and the underlying initial pressure rise distribution,^17^ significantly speeding up and improving the reconstruction process.

**Fig. 1:**
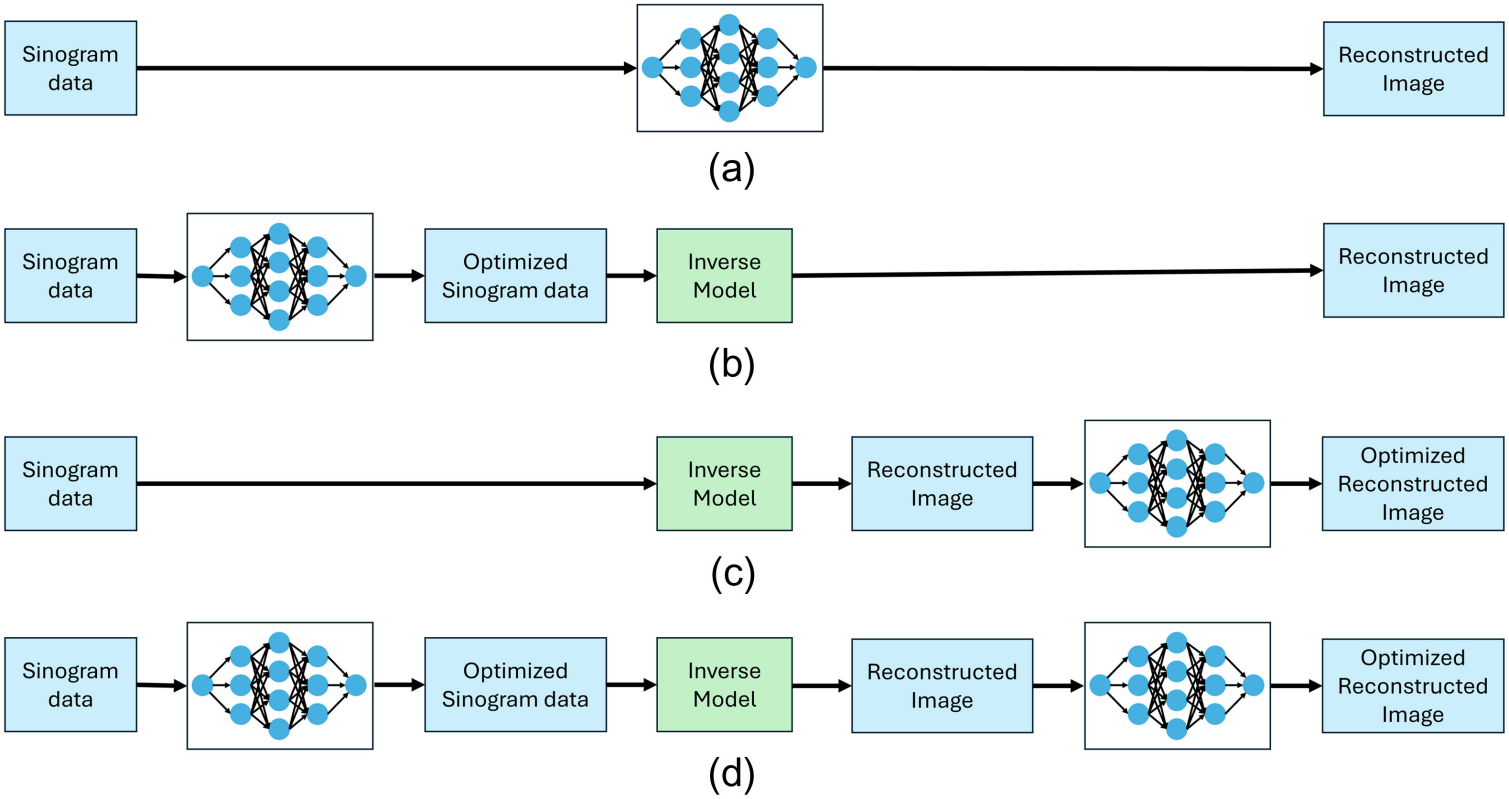
Overview of different deep-learning-based image reconstructions. (a) Direct deep-learning-based reconstruction: transform the sinogram data directly to the reconstructed image. (b) Indirect sinogram-based deep learning method: improve the sinogram data and further reconstruct the image using imverse model. (c) Indirect image-based deep-learning method: post-process the reconstructed image obtained after inversion. (d) Indirect deep-learning method: improve the sinogram data as well as the reconstructed image.

The deep-learning-based image reconstruction methods can be classified into four categories, as shown in Fig. 1. The first category^18^^–^^20^ involves developing deep neural network architecture having the ability to directly map the sinogram data to the reconstructed image, as illustrated in Fig. 1(a). Figures 1(b) and 1(d) represent the utility of deep learning approaches to improve the sinogram data [Fig. 1(b)], or the reconstructed image [Fig. 1(c)], or both [Fig. 1(d)].^16^^,^^21^^–^^23^ Along with an appropriate inverse model, these improvements enabled accurate PAI. Several deep learning techniques have been proposed, including convolutional neural networks (CNNs),^16^^,^^22^^,^^23^ recurrent neural networks (RNNs),^24^^,^^25^ and generative adversarial networks (GANs).^21^ CNNs are well suited for processing grid-like data such as images and can efficiently learn spatial features from recorded acoustic data. Importantly, CNNs may have difficulty while capturing long-range dependencies, i.e., not being able to recognize the pattern across the imaging domain (or time-series), which can affect reconstruction accuracy, and these algorithms tend to have longer training times with large datasets.^26^ RNNs are capable of capturing temporal dependencies in the PA data, making them useful for dynamic PAI applications, but RNNs may struggle to learn spatial features and need longer time for training with long sequences of data.^20^^,^^27^ GANs, on the other hand, can produce high-quality images by learning complex data distributions, making them suitable for PA image reconstruction, but they can be challenging to train and are often sensitive to hyperparameters.^28^ Transformers, which excel at capturing long-range dependencies, are more efficient than RNNs for image reconstruction, but like GANs, they can be computationally expensive and require careful tuning of hyperparameters.^29^^,^^30^ Hybrid approaches that combine different deep learning architectures have also been explored, such as using CNNs to extract spatial features and transformers to model long-range dependencies.^31^^–^^33^

SwinUNet, a U-Net-like transformer-based architecture, has demonstrated excellent performance in image segmentation tasks and is more efficient than traditional transformer models,^31^ primarily due to its use of hierarchical Swin Transformer blocks with shifted windows. This design enables localized self-attention and reduces computational overhead compared with global attention mechanisms. It has been shown earlier that the Swin architecture is efficient in terms of computational complexity and speed over vision transformers (ViT) while achieving superior accuracy across multiple vision tasks.^34^

By adapting SwinUNet for PA image reconstruction task, we aim to take advantage of the transformer’s ability to capture long-range dependencies and the U-Net’s potential while handling the two-dimensional imaging domain. However, like other transformer models, SwinUNet can be computationally expensive for large datasets and requires careful hyperparameter tuning. Furthermore, contrastive learning could improve image reconstruction by enhancing the quality of representations learned from noisy data. This approach leverages self-supervised learning to discern meaningful patterns without requiring extensive labeled datasets (a common challenge in medical imaging). Using contrastive loss functions, such as residual-based contrastive learning, the method emphasizes similarities between augmented versions of the same data while distinguishing them from other instances, enabling robust feature extraction even in noisy conditions.^35^^,^^36^ We hypothesize that developing contrastive learning approaches alongside SwinUNet architecture (based on transformer and UNet) would enable accurate photoacoustic image reconstruction under different imaging conditions.

Although contrastive objectives can be defined in either the image or sinogram domains, we use the contrastive loss in the sinogram (projection) domain in this work. In tomographic imaging, the sinogram domain represents the raw measurement space, where the forward model is linear and directly governed by the acquisition physics. One of the primary reasons for using sinogram-domain contrastive loss is that subtle differences between reconstructions are more prominent in sinograms than in reconstructed images as these differences often get masked or averaged out during the image reconstruction process. Enforcing similarity and dissimilarity in the sinogram domain ensures that the reconstructed images remain consistent with the measured data. Prior studies^37^ have shown that projection-space learning can preserve quantitative and structural information more effectively than purely image-space approaches under noisy or limited-view conditions, which motivates our sinogram-domain contrastive formulation for PAI.

The proposed Dual SwinUNet framework is built around three key components: a dual-network structure with two complementary reconstruction inputs, a sinogram-domain consistency term, and contrastive learning with positive and negative pairs. The dual-network structure simultaneously processes two images reconstructed using different analytical-model-based algorithms, such as backprojection and Tikhonov regularization, allowing the network to exploit complementary reconstruction strengths and reduce algorithm-specific artifacts that are pronounced under limited-view, sparse-sampling, and low-SNR conditions. The sinogram-domain loss couples the network outputs to the measurement space through the acoustic forward model, explicitly enforcing data consistency and reducing hallucinated structures that may arise when learning solely in the image domain. Finally, contrastive learning on positive and negative pairs in the sinogram domain encourages the model to learn representations that are consistent across reconstructions of the same structure while remaining discriminative across different structures, thereby improving robustness to noise and reconstruction ambiguity in PAT. Together, these components are intended to address the main failure modes of PAT reconstruction, including loss of vasculature, limited-view artifacts, and noise-induced inconsistencies, by combining complementary image priors, physics-based consistency, and discriminative representation learning within a single framework.

This paper introduces a PA image reconstruction method based on a dual SwinUNet architecture, designed to extract features from both the image and sinogram domains for enhanced performance. The key contributions of this work are as follows: (a) we propose a dual SwinUNet architecture that extracts features from both domains by utilizing two different reconstructed images (from different reconstruction algorithms) as inputs, (b) the dual SwinUNet architecture was compared with standalone SwinUNet architecture, (c) the dual SwinUNet architecture was evaluated in terms of accuracy and computational complexity compared with other deep-learning-based approaches at various noise levels (20, 30, and 40 dB SNR) and different transducer setups (40 and 100 transducers) as well as limited-view scenarios with coverage angles of 70 deg and 135 deg, and (d) dual SwinUNet and other approaches were compared with both synthetic and real *in vivo* mice PAI datasets with different DL models, i.e., ResNet, UNet, FDUNet, TNet, and the proposed dual SwinUNet model. Structural similarity index (SSIM), universal image quality index (UIQI), and generalized contrast-to-noise ratio (gCNR) were used as quantitative evaluation metrics.

## 2 Methods

### 2.1 Acoustic Forward Model

The acoustic forward model in PAI describes the relationship between the initial pressure rise in a tissue and the recorded PA signal. The acoustic forward model can be expressed using the wave equation,^38^^,^^39^

$$\frac{\partial^{2}p(\overset{\to }{r},t)}{\partial t^{2}}-v(\overset{\to }{r}{)}^{2}\nabla^{2}p(\overset{\to }{r},t)=H(\overset{\to }{r},t),$$ (1)

where $p(\overset{\to }{r},t)$ indicates the pressure rise at a spatial location $\vec{r}$ and at given time t, $v(\overset{\to }{r})$ represents the speed of sound in the medium, and $H(\overset{\to }{r},t)$ is the photoacoustic source term, which represents the initial acoustic pressure rise generated by the absorption of laser energy. The PA source term $H(\overset{\to }{r},t)$ is related to the optical absorption distribution $\mu_{a}(\vec{r})$ and the laser pulse energy E(t),

$$H\left(\overset{\to }{r},t\right)=\Gamma \left(\overset{\to }{r}\right)\mu_{a}(\overset{\to }{r})E(t),$$ (2)

where $\Gamma (\overset{\to }{r})$ is the Grüneisen parameter, which relates the absorbed optical energy to the generated acoustic pressure.

### 2.2 Image Reconstruction

By solving the acoustic and optical inverse problem,^40^ one can reconstruct the optical absorption distribution of the tissue from the measured PA data. The acoustic inverse problem involves estimating the initial pressure rise distribution from the recorded photoacoustic signals at various transducer locations. The acoustic forward model can be expressed as a linear process by,^6^^,^^40^

y=Ax, (3)

where A is the system/forward model matrix, y is the recorded acoustic signals, and x is the image that has to be reconstructed. In this work, one of the reconstructed images (for the contrastive-learning-based dual SwinUNet architecture) was obtained using backprojection approach, given as,

$$x_{bp}=A^{T}b.$$ (4)

The generalized objective function for different model-based reconstructions such as $\ell_{2}$-norm (Tikhonov-regularized)-based reconstruction^41^ and Cauchy-based penalization^42^ can be written as,^8^^,^^43^

$$\Omega =\frac{1}{2}\|b-Ax{\|}_{2}^{2}+\lambda \tau (x),$$ (5)

where τ(x) denotes the penalty function corresponding to different model-based reconstructions. The closed form solution with $\ell_{2}$-norm constrained PA image reconstruction is,^41^

$$x_{\mathrm{tikh}}=(A^{T}A+\lambda I{)}^{-1}A^{T}b.$$ (6)

The reconstructed PA image using the Cauchy-based penalization is given by,^42^

$$x_{\mathrm{cauchy}}=(A^{T}A+\lambda W{)}^{-1}A^{T}b,$$ (7)

where $W=\frac{1}{\left[\sigma_{x}^{2}+{x_{bp}}^{2}\right]}$ and $\sigma_{x}$ is the variance of $x_{bp}$. These reconstructed images were used as input for training contrastive-learning-based dual SwinUNet architecture.

### 2.3 Deep Learning Network

**Fig. 2:**
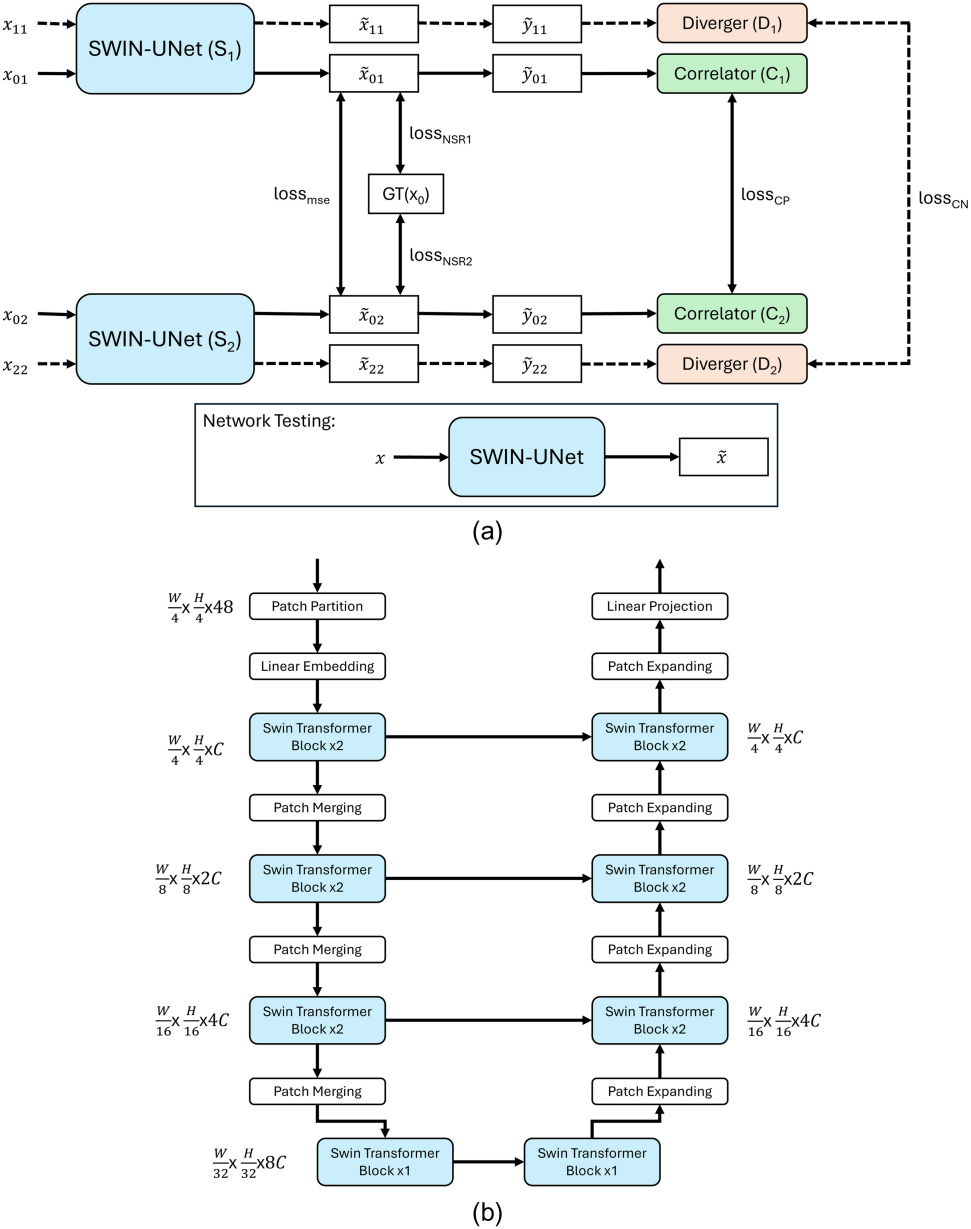
(a) Proposed architecture of dual SwinUNet. Here, $S_{1}$ and $S_{2}$ are two SwinUNet models fed with positive and negative pairs of reconstructed images using two reconstruction algorithms. ($x_{01}$ , $x_{02}$) is the positive pair, and ($x_{11}$ , $x_{22}$) is the negative pair. The forward model is applied on the network outputs ($\overset{˜}{x}$), and the resulted sinogram data ($\overset{˜}{y}$) are fed to divergers (D) and correlators (C). After training, the SwinUNet is used to evaluate the network performance, which is indicated by network testing block. (b) The SwinUNet model used in the proposed architecture.

The proposed network architecture consists of two SwinUNet networks ($S_{1}$ and $S_{2}$), as shown in Fig. 2(a). SwinUNet is built using Swin transformer blocks,^31^ as shown in Fig. 2(b), which leverage the transformer architecture’s window-based multihead self-attention mechanism to reduce computational complexity. This approach divides the input image into nonoverlapping windows and applies self-attention within each window, allowing the network to capture local dependencies while maintaining the global context. To merge information from different layers, cross-attention modules were incorporated between the encoder and decoder. These modules improve decoder learning by providing the features from corresponding encoder layers, facilitating effective integration of multiscale information. In the encoder, a combination of convolutional layers and successive down-sampling layers was utilized to capture deep features with broad receptive fields, while the decoder reverses this process, by upsampling feature maps with small receptive fields. This design ensures that the network can efficiently process and interpret information at various resolutions.

**Fig. 3:**
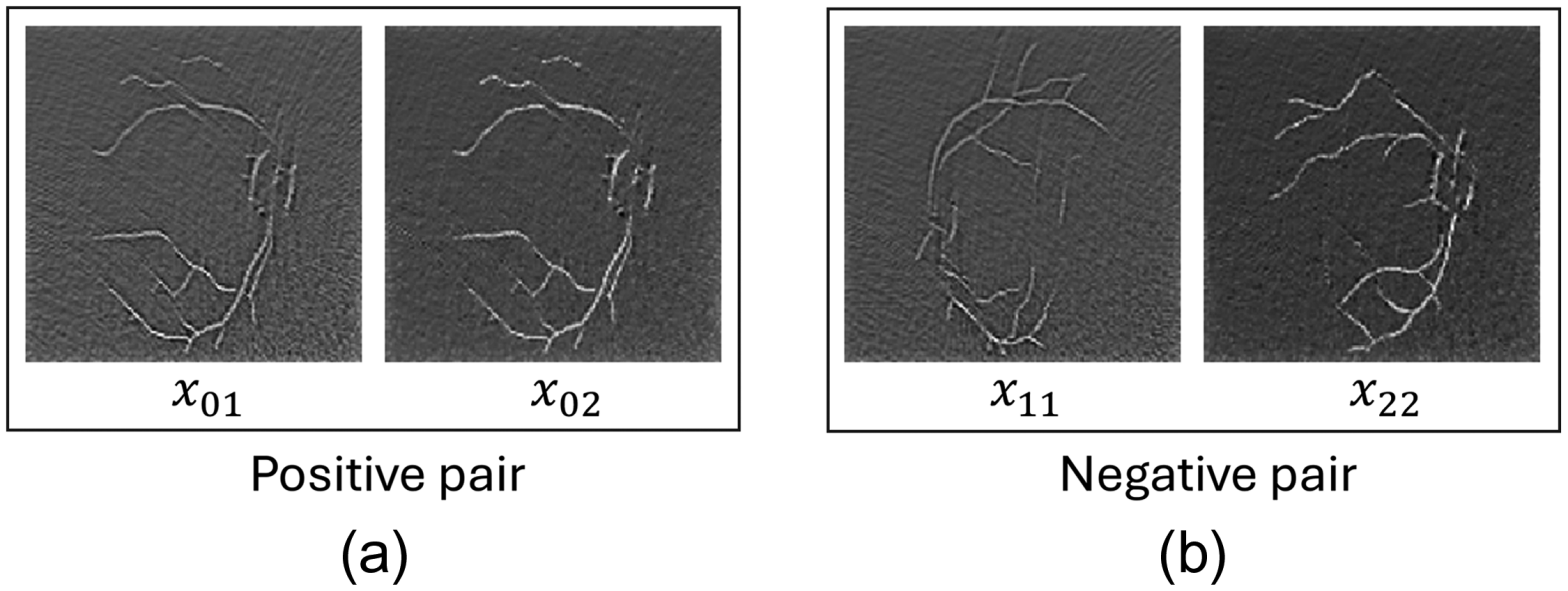
(a) Positive pair and (b) negative pair of the reconstructed images.

In the proposed dual SwinUNet network, the reconstructed image pair ($x_{i1}$, $x_{i2}$) from two different reconstruction algorithms, i.e., backprojection–Tikhonov, backprojection–Cauchy, or Tikhonov–Cauchy penalized reconstruction algorithm, were used as the input to each of the SwinUNet, named as $S_{1}$ and $S_{2}$, respectively. The network was trained using both positive and negative input pairs simultaneously, as shown in Fig. 3. Positive pairs refer to inputs that share the same ground truth, whereas negative pairs correspond to inputs with different ground truths. The mean squared error loss ($L_{\mathrm{mse}}$) is computed between the predicted output for the positive pair of the SwinUNet networks.^27^^,^^44^ The noise-to-signal ratio is also computed between the predicted output of the positive pair of the networks and their respective ground truth, which are named as ($L_{\mathrm{NSR}1}$, corresponding to $S_{1}$), and ($L_{\mathrm{NSR}2}$ corresponding to $S_{2}$).^45^^,^^46^

Although positive pairs encourage the network to produce consistent reconstructions across different forward-model inputs of the same structure, negative pairs from different images force the latent features to remain separable across anatomically distinct samples. This contrast helps the network preserve fine object-specific geometric details rather than converging to a generic smooth representation.

Furthermore, the acoustic forward operator is applied to the output of the SwinUNet model to transform the reconstructed image into the sinogram domain. The sinogram data of the negative pair are fed to the divergers^47^^,^^48^ to extract feature representation. Next, the dissimilarity between the two feature representations is measured using sample-wise contrastive loss ($L_{\mathrm{CL}}$), which is defined as,

$$L_{\text{CL}}=-\log\frac{\exp\left(q^{d}\cdot k_{+}^{d}/\tau \right)}{\exp\left(q^{d}\cdot k_{+}^{d}/\tau \right)+\overset{K}{\underset{i=0}{\sum }}\exp\left(q^{d}\cdot k_{i}^{d}/\tau \right)},$$ (8)

where $q^{d}$ and $k^{d}$ are the feature maps from the two divergers. $q^{d}\cdot k_{+}^{d}$ corresponds to the positive sample pair, $q^{d}\cdot k_{i}^{d}$ corresponds to the negative sample pair, and τ is the temperature constant (which is set to 0.07).

The divergers consider only negative pairs, hence $q^{d}\cdot k_{+}^{d}=0$. The contrastive loss for the negative pairs is given as,

$$L_{\text{CN}}=-\log\frac{\exp(0/\tau )}{\exp(0/\tau )+\overset{K}{\underset{i=0}{\sum }}\exp\left(q^{d}\cdot k_{i}^{d}/\tau \right)}=-\log\frac{1}{1+\overset{K}{\underset{i=0}{\sum }}\exp\left(q^{d}\cdot k_{i}^{d}/\tau \right)}$$ (9)

Furthermore, the sinogram data of the positive pairs are fed to the correlators^47^^,^^48^ to generate high-level feature maps. A contrastive loss ($L_{\mathrm{CP}}$) was used to measure the similarity of the two high-level feature maps, which is defined as,

$$L_{\text{CP}}=-\log\frac{\exp\left(r^{c}\cdot s_{+}^{c}/\tau \right)}{\exp\left(r^{c}\cdot s_{+}^{c}/\tau \right)+\overset{K}{\underset{i=0}{\sum }}\exp\left(r^{c}\cdot s_{i}^{c}/\tau \right)},$$ (10)

where $r^{c}$ and $s^{c}$ are the feature maps from the two correlators. $r^{c}\cdot s_{+}^{c}$ corresponds to the positive sample pair, $r^{c}\cdot s_{i}^{c}$ is the negative sample pair, and τ=0.07 is the temperature constant.

In all experiments, the temperature parameter in the contrastive losses $L_{\mathrm{CN}}$ and $L_{\mathrm{CP}}$ was fixed to τ=0.07, which provided stable training and good empirical performance, in line with prior contrastive learning work.^49^ The diverger and correlator branches play complementary roles in the sinogram domain: divergers, trained with $L_{\mathrm{CN}}$, enforce separation between features from different ground truths, whereas correlators, trained with $L_{\mathrm{CP}}$, promote invariance between reconstructions of the same ground truth obtained with different algorithms. Using both modules allows the network to jointly push negatives apart and pull positives together, yielding more informative and physically consistent sinogram-space embeddings.

For network training, 1:1 negative sampling strategy was used, i.e., for each positive pair, exactly one negative pair was selected randomly from the minibatch with a different ground truth. This choice balances the benefits of contrastive supervision with the computational and memory overhead of the dual-network architecture, which already doubles the number of forward and backward passes.

In addition, the embeddings and feature vectors are normalized, prior to computing dot product similarity. This normalization ensures that the similarity scores are bounded and scale-invariant, which is critical for stable training and meaningful contrastive comparisons. Finally, both the network parameters are updated simultaneously by the sum of these five losses:

$$L_{\mathrm{total}}=w_{1}*L_{\mathrm{mse}}+w_{2}*L_{\mathrm{NSR}1}+w_{3}*L_{\mathrm{NSR}2}+w_{4}*L_{\mathrm{CN}}+w_{5}*L_{\mathrm{CP}}.$$ (11)

Here, $w_{1}⏧w_{5}$ are the weights for each loss; all the weights were set as 1/5. The dual Swin-UNet model is developed in PyTorch. A Linux workstation with Intel Core i9-10900X CPU and NVIDIA graphics card RTX A6000 having a speed of 3.70 GHz with 128 GB RAM was used to train the models. The network was trained with numerical dataset for 600 epochs, using Adam optimizer ($\beta_{1}=0.9$ and $\beta_{2}=0.999$) with the varying learning rate as:

$$\mathrm{lr}={\mathrm{lr}}_{i}*\left(1-\frac{\mathrm{epoch}}{{\mathrm{epoch}}_{\mathrm{tot}}}\right)**0.9.$$ (12)

Here, ${\mathrm{lr}}_{i}$ is the initial learning rate, which is set at $1\times 10^{-4}$, epoch is the current epoch, and ${\mathrm{epoch}}_{\mathrm{tot}}$ is the total number of epochs. Furthermore, to evaluate the network performance with the experimental dataset (*in vivo* data), the pre-trained network is finetuned for 40 epochs. The *in vivo* dataset consisted of 20 mice, with 30 scans acquired per mouse. The proposed dual SwinUNet was trained in two stages. First, the network was pre-trained for 600 epochs on simulated vasculature images generated from the RFMiD fundus dataset after preprocessing and PAT-based forward simulation, enabling the network to learn general reconstruction priors, vascular structures, and measurement-to-image mapping in PAT. Second, the pre-trained model was fine-tuned on 450 *in vivo* mouse slices for 40 epochs. For the *in vivo* study, the experimental configuration is similar to the simulation geometry used during pre-training, so fine-tuning the pre-trained Dual SwinUNet on the *in vivo* data is sufficient. In addition, longer fine-tuning did not improve performance and increased overfitting risk. On the other hand, for the phantom study, the acquisition geometry differed substantially from the simulation setup; therefore, geometry-matched phantom-like simulated data were generated and used to retrain the model before evaluation on the real phantom measurements. Although transformer architectures typically benefit from larger datasets, large-scale annotated *in vivo* PAT datasets are currently limited in the public domain. Hence, this two-stage training strategy offers a practical way to apply transformer-based reconstruction to photoacoustic imaging with limited *in vivo* data. More details about the dataset used for training and testing of the network are given in Table 1.

**Table 1:** Dataset information for the training, validation, and testing of the network.

Dataset	Training data	Validation data	Testing data
RFMiD fundus dataset	2573	300	100
*In vivo* mice imaging dataset	450	60	90

### 2.4 Simulated Data

First, the RFMiD Fundus data were resized to 256×256  pixels and then converted into a grayscale image. To enhance the contrast of the blood vessels, the contrast-limited adaptive histogram equalization algorithm was applied. Next, image smoothing was performed to improve overall image quality, followed by thresholding and morphological operations to create a binary image. In addition, to make the phantom comparable to a realistic numerical phantom, background absorption values were added. The final image obtained from this process served as the ground truth image. Herein, we used three different transducer setups, specifically a spherical concave array transducer configuration with three combinations of 40 and 100 point detectors, and angular coverage of 70 deg and 135 deg. These configurations had a focus at 37 mm, and a radius of curvature of 40 mm.

For all transducer configurations, the number of time samples, n, was based on the distance between the transducer channel and the acoustic source. The sampling rate was considered as 40 MHz. The imaging region was set to 256×256  pixels, making the size of the forward model matrix A equal to NM × NN, where NM is the number of transducers (either 40 or 100) × the number of time samples (1500), and NN for the forward model was 256×256=65,536. To avoid inverse crime, a coarser resolution was used for the inverse model, with the image size reduced to 128×128  pixels, resulting in NN being 128×128=16,384. Simulations were performed with three different noise levels corresponding to data signal-to-noise ratio (SNR) values of 20, 30, and 40 dB. Image reconstruction was carried out using four different reconstruction algorithms: time-reversal,^50^ backprojection,^51^ Tikhonov regularization,^41^ and Cauchy-based penalized reconstruction.^42^

The RFMiD Fundus dataset does not fully capture the three-dimensional absorption and scattering heterogeneity of bulk tissue in PAT. However, it provides a large number of high-resolution vascular patterns that are useful for learning structural priors relevant to two-dimensional PA reconstruction. In this work, the fundus images were converted to grayscale, preprocessed, and binarized, after which homogeneous background absorption and PAT forward simulation were applied to generate numerical training data. Thus, the network was trained on PA-like measurements rather than raw optical images. Training directly on vascular PA datasets would be a natural extension of this work, but large paired datasets with controlled geometry are currently limited.

### 2.5 Phantom Preparation

The tissue-mimicking phantom was prepared by dissolving 1.2 g of agar to 100 ml of hot deionized water under continuous stirring until the solution become homogeneous. Furthermore, intralipid (6% by volume) was added as a scattering agent, and the solution was stirred thoroughly to ensure uniformity. The well mixed homogeneous solution was poured into the mold and allowed to solidify. Following solidification, the graphite rods, serving as absorbers, were inserted at the desired points within the phantom. Three phantoms were prepared, containing 1, 3, and 5 graphite rods, respectively, with the rods positioned at different locations in each phantom.

### 2.6 Experimental Dataset

It is important to note that the simulated data acquisition setup was different from the phantom experimental configuration. Consequently, the deep learning models were retrained with the simulated data corresponding to the experimental setup used for phantom data collection. The resulting model was used to validate the phantom measurements.

**Fig. 4:**
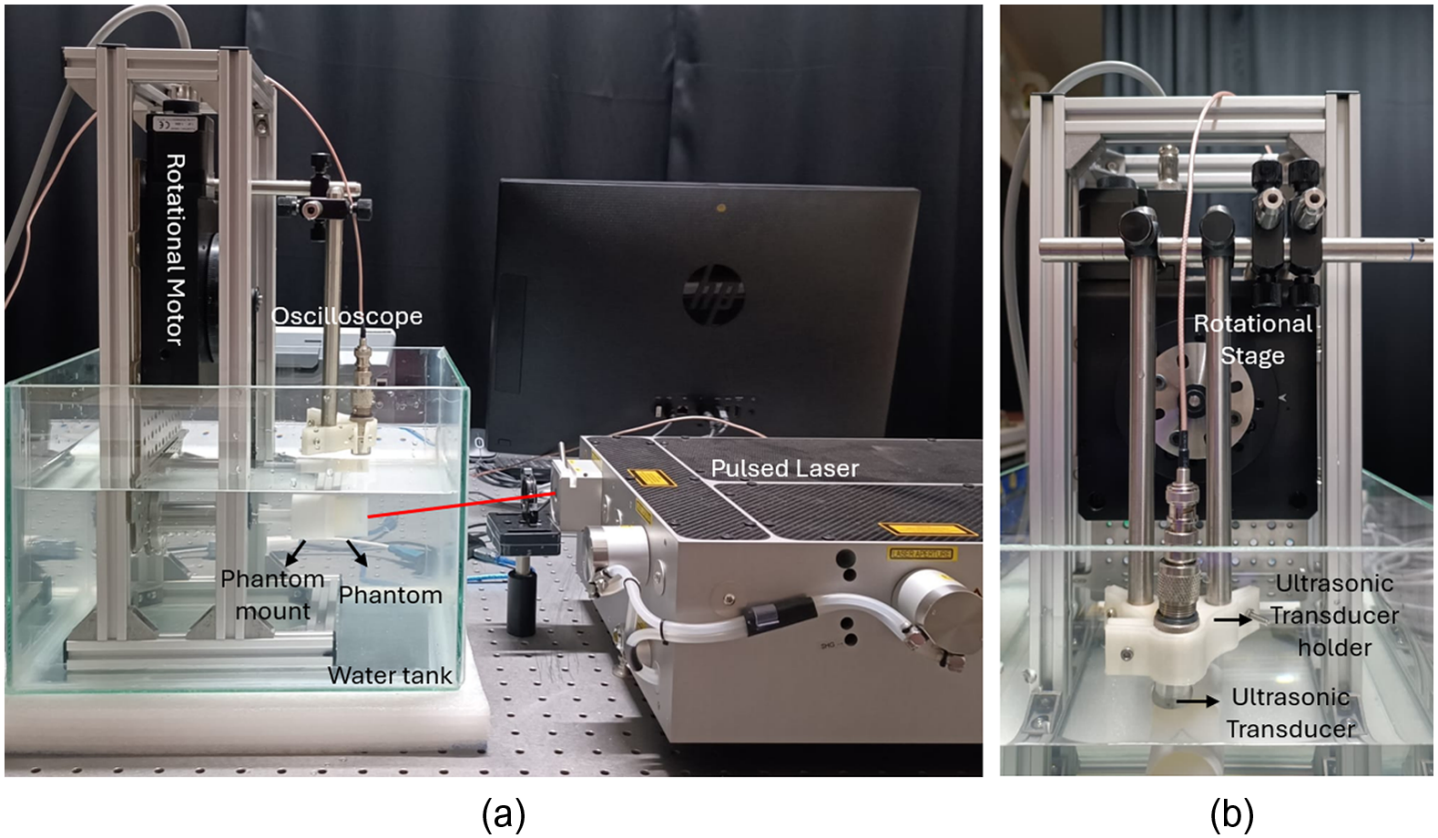
(a) Photograph of the photoacoustic imaging experimental set up. (b) Front view of the sample illumination with the transducer placed on the top of the sample.

#### 2.6.1 In silico data

The schematic of the experimental set up is shown in Fig. 4. A nanosecond Nd:YAG pulsed laser (INNOLAS SpitLight 1000 OPO-532) with a tunable wavelength range of 660 to 2500 nm having a pulse width of 7 ns and operating at a repetition rate of 30 Hz was used to irradiate the tissue-mimicking phantom. As the graphite was used as the absorber, the illumination wavelength was set to 830 nm, where graphite exhibits peak optical absorption, with an average energy of 18 mJ. A single-element unfocused ultrasound transducer (Olympus V309-SU, Hachioji, Japan) having a center frequency of 5 MHz and a bandwidth of 61% was used as a detector. The transducer signal was digitized using a digital storage oscilloscope (RIGOL MSO2102A, Suzhou, China) operating at a sampling frequency of 20 MHz, and the acquisition was synchronized with the laser pulse trigger. Herein, the phantom was mounted on a motorized rotational stage (Holmarc HO-MPC-1H, Kochi, India) and rotated through a full 360 deg to enable complete angular coverage. Acoustic signals were recorded at 200 uniformly distributed angular positions around the phantom, ensuring comprehensive data acquisition for image reconstruction. Following the acquisition of the photoacoustic signals, the data were post-processed using an 8th-order Chebyshev type-1 bandpass filter with a cutoff frequency of [0.1 MHz, 8 MHz]. The filtered signals were then used for image reconstruction via the back-projection algorithm, followed by contrast stretching to enhance image quality.

#### 2.6.2 In vivo mice data

*In vivo* imaging data from mice were acquired using a commercial MSOT scanner (MSOT256-TF, iThera Medical GmbH, Munich, Germany). Nanosecond-pulsed light was generated by a tunable optical parametric oscillator (OPO) laser [600 to 900 nm] and delivered to the sample via a ring-type fiber bundle having 10 outputs. The acquisition involved 10 averages at each wavelength. The absorbed light generated acoustic signals, which propagated through the sample and were detected externally. The time-series acoustic signals were acquired at 2030 discrete time points, sampled at 40 MHz, using a cylindrically focused transducer array consisting of 256 elements. The transducer had a central frequency of 5 MHz (−6  dB bandwidth of ∼90%) with a 40 mm radius of curvature and 270 deg angular coverage. Acoustic data were collected across multiple laser wavelengths and were filtered using a Chebyshev filter with cut-off frequencies between 0.1 and 7 MHz. PA images were reconstructed using Chebyshev filtered PA data with backprojection algorithm and Tikhonov regularization techniques. The reconstructed image with full data was used as the ground truth. The sinogram data from different transducer channels were selected to perform reconstruction for the case with 100 transducers having 135 deg angular coverage. All mice procedures were performed in accordance with appropriate standards under protocols approved by the Animal Care and Use Committee of the First Affiliated Hospital, Zhejiang University School of Medicine (2023-986).

### 2.7 Evaluation Metrics

To quantitatively evaluate the performance of different networks i.e., ResNet,^52^ UNet,^53^ FDUNet,^16^ TNet,^54^ and the proposed model, three figure of merits were computed:

#### 2.7.1 Structural similarity index measure (SSIM)

SSIM is the measure of the similarity of two images, i.e., predicted output and the ground truth image in terms of structural details, which is given as

$$\mathrm{SSIM}=\frac{\left(2\mu_{\overset{˜}{x}}\mu_{x}+c_{1}\right)\left(2\sigma_{\overset{˜}{x}x}+c_{2}\right)}{\left(\mu_{\overset{˜}{x}}^{2}+\mu_{x}^{2}+c_{1}\right)\left(\sigma_{\overset{˜}{x}}^{2}+\sigma_{x}^{2}+c_{2}\right)},$$ (13)

Here, $\overset{˜}{x}$ and x are the predicted output image and ground truth image, and μ and σ represent the mean and the standard deviation of the image.^55^ The range for the SSIM values are [0, 1], and higher value of SSIM indicates the higher accuracy and better image quality of the output.

#### 2.7.2 Universal image quality index (UIQI)

UIQI is a metric used to assess the perceptual quality of images. It is designed to be sensitive to both local and global image distortions, and to correlate well with human subjective judgments of image quality. The UIQI is calculated using the following equation:^56^

$$\mathrm{UIQI}=\frac{\left(4\mu_{I_{1}}\mu_{I_{2}}\sigma (I_{1},I_{2})\right)}{\left(\sigma_{I_{1}}^{2}+\sigma_{I_{2}}^{2}\right)\left(\mu_{I_{1}}^{2}+\mu_{I_{2}}^{2}\right)}.$$ (14)

Here, $I_{1}$ and $I_{2}$ are the predicted image and the ground truth image, and μ and σ represent the mean and the covariance of the image, and, $\sigma^{2}$ represents the variance of the image. The UIQI ranges from −1 to 1. The value of 1 represents the perfect-quality image, and −1 represents the worst possible image quality.

#### 2.7.3 Generalized contrast-to-noise ratio (gCNR)

The generalized contrast-to-noise ratio (gCNR) is a image quality metric introduced in Ref. 57. This metric offers a more robust and accurate assessment of medical images compared with traditional metrics such as the contrast-to-noise ratio (CNR). Unlike CNR, gCNR is not affected by changes in dynamic range, making it more reliable for evaluating image quality in various imaging modalities. The gCNR is calculated based on the overlap between the probability density functions of the background and foreground regions in the image. The gCNR can be calculated as

$$\mathrm{gCNR}=1-2P_{\min}=1-\mathrm{OVL},$$ (15)

where $P_{\min}$ is the minimum probability error, and OVL is the overlap area between both the probability density function, i.e., probability density function of the values taken by the foreground and background pixels.

## 3 Results

### 3.1 Effect of Number of Transducers

**Fig. 5:**
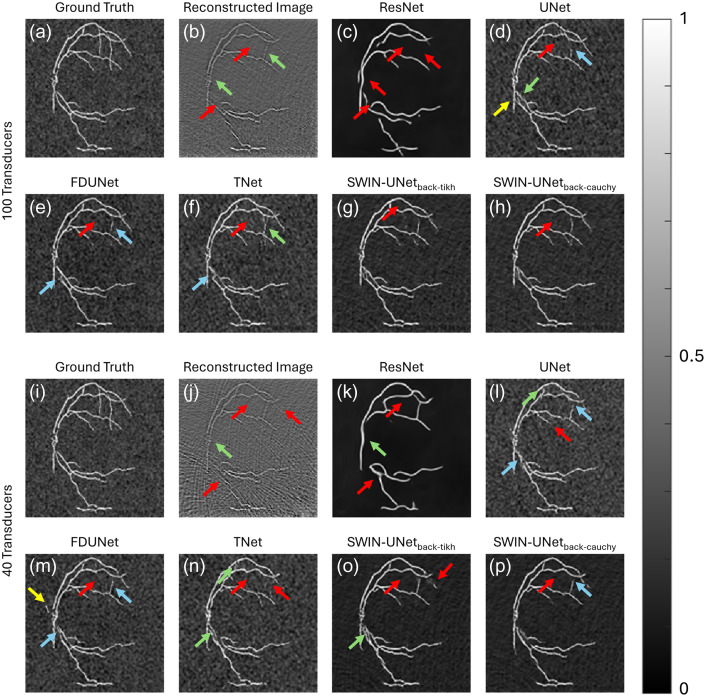
Effect of number of transducers having a coverage of 135 deg with different reconstruction models. Here, panels (a)–(h) and (i)–(p) represent the ground truth, backprojection output, and outputs from different deep learning models for 100 and 40 transducers, respectively. The blue and green arrows represent the distorted/discontinuous and blurred structures, respectively. The structures that have not been recovered and false structures are represented by red and yellow arrows, respectively.

The reconstructed images corresponding to 40 and 100 transducers, along with the respective deep learning (DL) outputs, are depicted in Fig. 5. Figures 5(a) and 5(b) represent the ground truth and the reconstructed image obtained using the backprojection algorithm. The backprojection output was found to introduce noisy/grainy texture in the background, along with a reduction in the image contrast while using 100-transducer acquisition. The small structures in the reconstructed image appeared to be discontinuous, and few structures were blurred; these were highlighted by red and green arrows in Fig. 5(b). Figures 5(c)–5(f) represent the predicted outputs of ResNet, UNet, FDUNet, and TNet, respectively. Due to the absence of skip connections between the encoder and decoder, ResNet failed to recover many structures and has smoothed the entire background, highlighted by red-colored arrows in Fig. 5(c). The UNet model incorporated skip connections and the FDUNet architecture had an additional fully dense layer for each spatial dimension; hence, the performance of these two networks (in terms of structure and background recovery) was better than the ResNet architecture. However, there are a few small structures that were not recovered using UNet and FDUNet architectures [highlighted by red arrow in Figs. 5(d) and 5(e)]. Furthermore, a few blurred and distorted structures were found in UNet and FDUNet output, which were indicated using green, and blue colored arrows in Figs. 5(d) and 5(e). The performance of TNet was similar to UNet, as shown in Fig. 5(f). The predicted output of the SwinUNet trained with backprojection and Tikhonov based reconstructed images, and backprojection and Cauchy-based reconstructed images were shown in Figs. 5(g)–5(h), respectively. The predicted images with SwinUNet were much closer to the ground truth image. Eventhough the SwinUNet model was not able to recover the fine structures (indicated with red arrow in Figs. 5(g)–5(h)), the reconstructed images were free from blurred or discontinuous structures. Quantitative metrics, including SSIM, UIQI, and gCNR, were used to assess network performance for varying numbers of transducers, as outlined in Table 2. The gCNR metric value for the ResNet is highest due to the absence of the background, with background pixel values predicted as zero. It is evident with the quantitative metrics that the UNet and TNet have the similar performance, whereas FDUNet performs slightly better than the UNet and TNet. Swin-UNet networks show improvements of 7.6%, 20%, and 12% in SSIM, UIQI, and gCNR, respectively, compared with the state-of-the-art FDUNet architecture for the 100-transducer cases having 40 dB data SNR.

**Table 2:** Quantitative comparison (using SSIM, UIQI, and gCNR metrics) of different image reconstruction algorithms/models with varied number of transducers (i.e., 100 and 40) having a coverage of 135 deg.

Network	100 transducers			40 transducers		
	SSIM	UIQI	gCNR	SSIM	UIQI	gCNR
Backprojection reconstructed image	0.502±0.053	0.520±0.042	0.307±0.010	0.383±0.037	0.354±0.029	0.221±0.014
Tikhonov reconstructed image	0.572±0.051	0.608±0.042	0.312±0.017	0.457±0.037	0.434±0.032	0.271±0.024
ResNet	0.414±0.074	0.206±0.075	0.615±0.025	0.392±0.087	0.181±0.067	0.596±0.027
UNet	0.779±0.068	0.618±0.060	0.334±0.008	0.668±0.091	0.438±0.082	0.311±0.006
FDUNet	0.795±0.067	0.645±0.054	0.336±0.006	0.686±0.093	0.456±0.079	0.338±0.006
TNet	0.778±0.069	0.623±0.059	0.334±0.006	0.668±0.091	0.439±0.081	0.314±0.008
${\mathrm{Swin}\text{-}\mathrm{UNet}}_{\mathrm{back}\text{-}\mathrm{tikh}}$	0.856±0.029	0.769±0.027	0.373±0.011	0.747±0.049	0.566±0.045	0.426±0.015
${\mathrm{Swin}\text{-}\mathrm{UNet}}_{\mathrm{back}\text{-}\mathrm{cauchy}}$	0.855±0.030	0.768±0.026	0.378±0.011	0.750±0.050	0.567±0.045	0.423±0.015

Note: bold values indicate the best performance for each evaluation metric across all methods evaluated.

When the number of transducers was reduced to 40, the backprojection reconstructed image exhibited increased noise/artifacts, and larger structures appeared blurry, as indicated with a green arrow in Fig. 5(j). The reconstruction algorithm was not able to recover most of the small and complex structures, as highlighted with the red arrows [in Fig. 5(j)]. Figures 5(k)–5(n) represent the predicted outputs of ResNet, UNet, FDUNet, and TNet, respectively. The performance of all four networks was similar to the 100-transducer case. The ResNet struggled to recover the structures as well as the background, as indicated by red arrows in Fig. 5(k). The performance of UNet, FDUNet, and TNet is similar, as depicted in Figs. 5(l)–5(n), with distorted, blurred features and unrecovered regions indicated by blue, green, and red arrows, respectively. The false structures are highlighted with yellow arrows. The performance of SwinUNet for both the input pair cases surpasses the other networks in terms of structure recovery and image contrast, as shown in Figs. 5(o) and 5(p). The SwinUNet output [Figs. 5(o) and 5(p)] does have a few missing blurred and distorted structures due to the reduction in the number of transducers; these are highlighted with red, blue, and green arrows, respectively. Quantitatively, the proposed network had improved image quality in terms of SSIM, UIQI, and gCNR metrics by 8.8%, 24%, and 26% over FDUNet (indicated in Table 2) with 40 dB data SNR, respectively.

### 3.2 Effect of Limited View

**Fig. 6:**
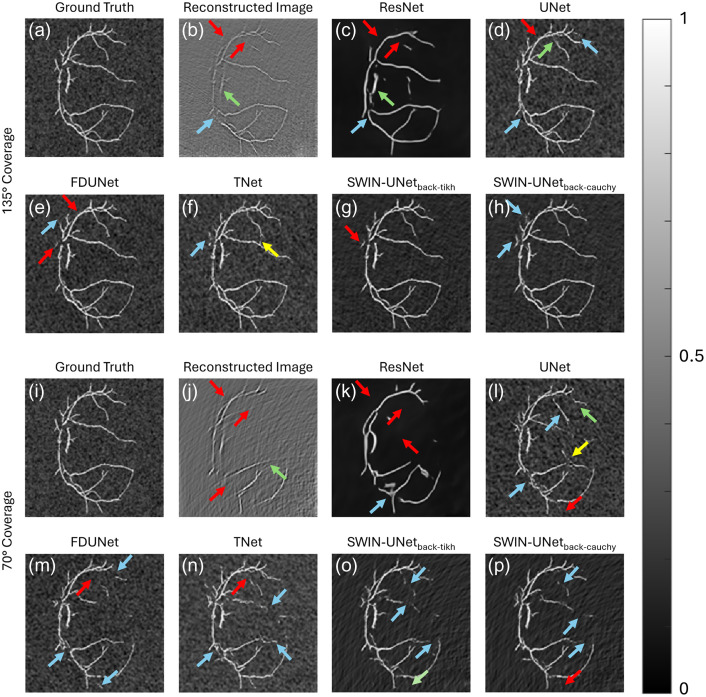
Effect of angular coverage with 100 transducers on different reconstruction algorithm/model performance. Panels (a)–(h) and (i)–(p) represent the ground truth, backprojection output, and the deep learning models outputs for 135 deg and 70 deg angular coverage, respectively. The blue and green arrows represent the distorted/discontinuous and blurred structures, respectively. The structures that have not been recovered and false structures are represented by red and yellow arrows, respectively.

The performance of various DL architectures was assessed for limited angular coverage scenarios, i.e., 135 deg and 70 deg. The reconstructed images corresponding to 70 deg and 135 deg coverage angle with 100 transducers, along with their respective DL improvements, are depicted in Fig. 6. Figures 6(a) and 6(b) represent the ground truth and the reconstructed image obtained using the backprojection reconstruction algorithm for 135 deg angular coverage. The backprojection image had reduced contrast, blurred, and distorted structures indicated using green and blue arrows in Fig. 6(b). Few structures were not recovered in the backprojection image, which are highlighted using red arrows in Fig. 6(b). The predicted outputs from different DL models (ResNet, UNet, FDUNet, TNet, and the proposed model) were compared. The ResNet architecture managed to recover the boundaries; however, finer structures and the background details were lost, as shown with red arrows in Fig. 6(c). Figures 6(d) and 6(e) illustrate the UNet and FDUNet outputs, which performed similarly in terms of retaining structural information and image contrast. However, few small structures were blurred and distorted (highlighted by green and blue arrows), and unrecovered structures were indicated by red arrows in Figs. 6(d) and 6(e). TNet’s performance was comparable to UNet, though some structures appeared distorted, as indicated by the yellow arrow in Fig. 6(f). Figures 6(g) and 6(h) indicate the predicted outputs of the proposed network trained with backprojection–Tikhonov, and backprojection–Cauchy reconstructed image pairs, respectively. The proposed network significantly improved the image contrast. However, the SwinUNet output with Tikhonov-regularized solution as an input was unable to recover one small structure, highlighted with the red arrow in Fig. 6(g), due to the smooth nature of the Tikhonov reconstructed images. Nevertheless, the Cauchy-based penalization focused on better structure recovery, allowing Fig. 6(h) to recover all structures with slight distortion highlighted with blue arrows. These findings aligned well with the quantitative metrics presented in Table 3.

**Table 3:** Quantitative metric comparison of various image reconstruction algorithm/deep learning model while using data acquired with 100 transducers having 40 dB SNR and varying angular coverage (135 deg and 70 deg).

Network	135 deg coverage			70 deg coverage		
	SSIM	UIQI	gCNR	SSIM	UIQI	gCNR
Backprojection reconstructed image	0.502±0.053	0.520±0.042	0.307±0.010	0.434±0.050	0.423±0.042	0.350±0.018
Tikhonov reconstructed image	0.572±0.051	0.608±0.042	0.312±0.017	0.434±0.050	0.423±0.042	0.352±0.011
ResNet	0.414±0.074	0.206±0.075	0.615±0.025	0.376±0.077	0.168±0.056	0.612±0.023
UNet	0.779±0.068	0.618±0.060	0.334±0.008	0.636±0.078	0.460±0.060	0.328±0.007
FDUNet	0.795±0.067	0.645±0.054	0.336±0.006	0.653±0.071	0.473±0.056	0.322±0.005
TNet	0.778±0.069	0.623±0.059	0.334±0.006	0.642±0.076	0.470±0.056	0.347±0.008
${\mathrm{Swin}\text{-}\mathrm{UNet}}_{\mathrm{back}\text{-}\mathrm{tikh}}$	0.856±0.029	0.769±0.027	0.373±0.011	0.716±0.051	0.597±0.034	0.371±0.007
${\mathrm{Swin}\text{-}\mathrm{UNet}}_{\mathrm{back}\text{-}\mathrm{cauchy}}$	0.855±0.030	0.768±0.026	0.378±0.011	0.718±0.058	0.595±0.038	0.368±0.009

Note: bold values indicate the best performance for each evaluation metric across all methods evaluated.

As the reconstructed images are normalized, vasculature regions with high recovered intensity may appear visually saturated even though their pixel values are not binary. Nonetheless, under the most challenging limited-view and noisy conditions, a small fraction of very fine vascular branches remain under-reconstructed, as indicated by the red arrows in Figs. 5 and 6.

For 70 deg angular coverage, the backprojection output had reduced image contrast and many structural artifacts due to decreased angular coverage; these artifacts are highlighted using red arrows in Fig. 6(j). The reconstructed image has distorted features highlighted by the green arrow in Fig. 6(j). The predicted outputs using ResNet, UNet, FDUNet, and TNet were shown in Figs. 6(k)–6(n). As in the previous cases, ResNet lost background information, and most finer features were indicated using red arrows in Fig. 6(k). The image contrast and the structures recovered using UNet and FDUNet were similar, with some smaller structures blurred (green arrows) and distorted (blue arrows), and the red arrow represents the unrecovered structures in Figs. 6(l)–6(m). The UNet architecture had introduced some false structures as well, indicated by the yellow arrow in Fig. 6(l). The performance of TNet was found to be similar to UNet in terms of image contrast; however, few structures had more distortions compared with UNet and FDUNet, as indicated with blue arrows in Fig. 6(n). Figures 6(o)–6(p) represent the predicted outputs of the proposed network for backprojection–Tikhonov and backprojection–Cauchy image pairs, respectively. The proposed network achieved sharper structure recovery and better image contrast, as compared to other standard models. Due to limited-view acquisition, some structures and the background were not recovered well even with the proposed network. The SwinUNet output with backprojection–Tikhonov pair had few distorted [blue arrows in Fig. 6(o)] and blurry structures, [green arrow in Fig. 6(o)]. In Fig. 6(p), the red arrow indicates a structure that was not recovered. Furthermore, the quantitative analysis using SSIM, UIQI, and gCNR metrics was shown in Table 3. The quantitative metrics demonstrated SwinUNet’s improvement of 9.2%, 27%, and 15%, respectively, compared with FDUNet with an angular coverage of 70 deg. The background of ResNet was zero, resulting in the highest gCNR value.

### 3.3 Effect of Noise

To simulate and test the performance of different networks, noise was added to the sinogram data to achieve three different data SNR levels, i.e., 20, 30, and 40 dB. Herein, image reconstruction was performed using backprojection, Tikhonov, and Cauchy-based penalization. All the networks were trained and tested with the data at each SNR level, and the quantitative results using SSIM, UIQI, and gCNR, were indicated in Table 4. Table 4 indicates that the values of the evaluation metrics, i.e., SSIM, UIQI, and gCNR, had increased with an increase in the data SNR. The performance of ResNet was degraded because of the absence of skip connections between the encoder and decoder. The performance of UNet and that of TNet were similar, whereas FDUNet had a slight improvement in terms of SSIM, UIQI, and gCNR, compared with UNet and TNet. The results (for UNet, FDUNet, and TNet) indicated significant improvement in terms of SSIM and UIQI with an increase in data SNR; however, gCNR varied marginally for these cases. The proposed SwinUNet architecture outperformed the other networks at all noise levels. As the data SNR increased, the performance of the proposed network had improved, i.e., for 40 dB data SNR case, an improvement of 7.5%, 18%, and 8.8% in SSIM, UIQI, and gCNR measures was observed compared with FDUNet. However, with a 20 dB data SNR, the improvement reduced to 6.7%, 16%, and 12% in SSIM, UIQI, and gCNR measures, respectively.

**Table 4:** Quantitative comparison (in terms of SSIM, UIQI, and gCNR) of backprojection reconstructed image and the DL model outputs using 100 transducers with the coverage of 135 deg at different noise levels. The network performance is tested for 20, 30, and 40 dB SNR.

SNR	Metrics	Network input	ResNet	UNet	FDUNet	TNet	${\mathrm{SwinUNet}}_{\mathrm{bp}\text{-}\mathrm{TR}}$	${\mathrm{SwinUNet}}_{\mathrm{bp}\text{-}\mathrm{tikh}}$	${\mathrm{SwinUNet}}_{\mathrm{bp}\text{-}\mathrm{cauchy}}$
20 dB	SSIM	0.44±0.04	0.40±0.08	0.71±0.10	0.74±0.09	0.72±0.10	0.79±0.06	0.80±0.05	0.79±0.05
	UIQI	0.42±0.04	0.19±0.07	0.51±0.08	0.54±0.08	0.52±0.08	0.61±0.06	0.64±0.06	0.63±0.06
	gCNR	0.24±0.01	0.75±0.01	0.33±0.01	0.33±0.01	0.32±0.01	0.35±0.01	0.37±0.01	0.38±0.01
30 dB	SSIM	0.50±0.05	0.42±0.08	0.77±0.07	0.79±0.07	0.77±0.07	0.84±0.04	0.85±0.03	0.85±0.03
	UIQI	0.51±0.04	0.21±0.08	0.61±0.07	0.63±0.06	0.61±0.06	0.72±0.03	0.75±0.03	0.75±0.03
	gCNR	0.29±0.01	0.63±0.02	0.33±0.01	0.33±0.01	0.33±0.01	0.35±0.01	0.38±0.01	0.38±0.01
40 dB	SSIM	0.50±0.05	0.41±0.07	0.78±0.07	0.80±0.07	0.78±0.07	0.85±0.04	0.86±0.03	0.86±0.03
	UIQI	0.52±0.04	0.21±0.08	0.62±0.06	0.65±0.05	0.62±0.06	0.74±0.03	0.77±0.03	0.77±0.03
	gCNR	0.31±0.01	0.62±0.03	0.33±0.01	0.34±0.01	0.33±0.01	0.35±0.01	0.37±0.01	0.38±0.01

Note: bold values indicate the best performance for each evaluation metric across all methods evaluated.

### 3.4 *In Silico* Imaging Data

**Fig. 7:**
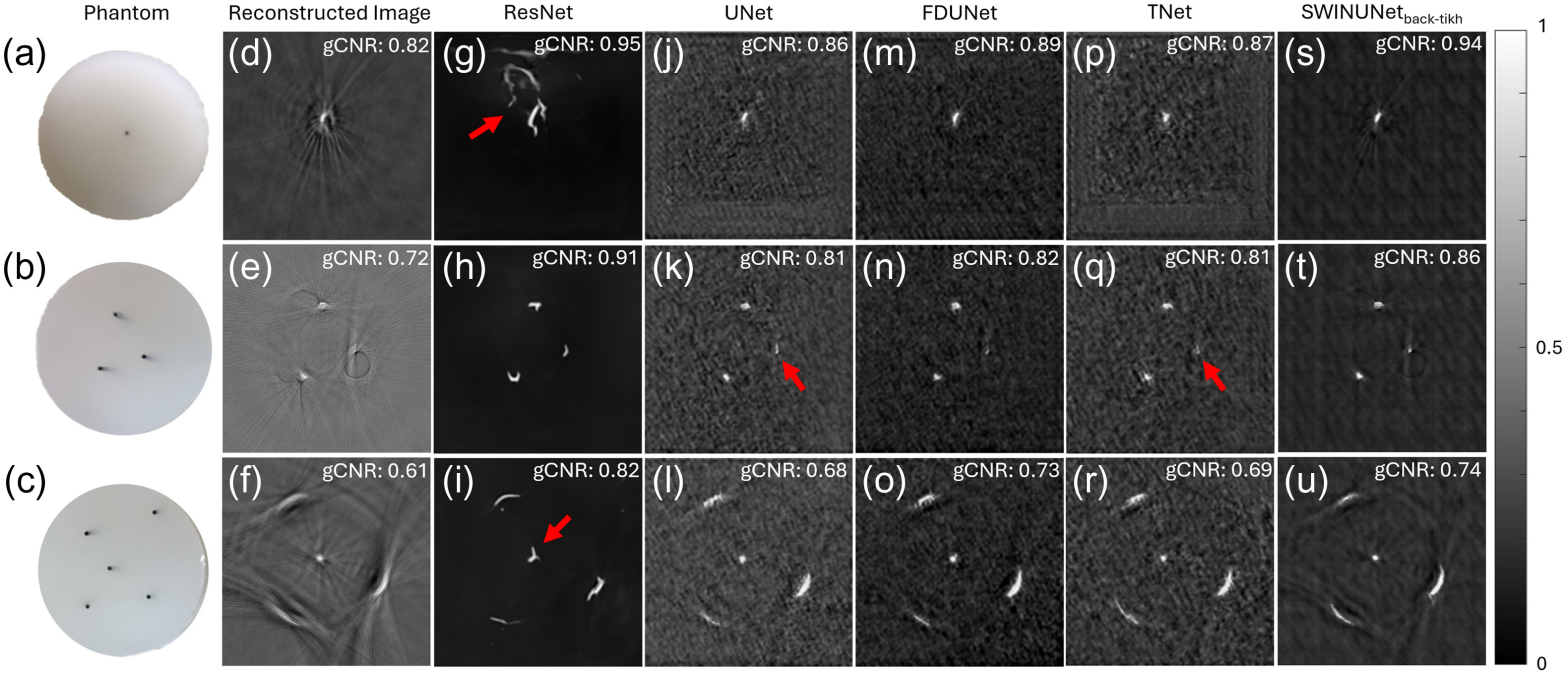
Phantom experiments with agar-based phantoms containing intralipid as scatterers. (a)–(c) Phantoms containing 1, 3, and 5 embedded rods embedded at different positions. (d)–(f) Reconstructed images obtained using the backprojection method, and post-processed images with (g)–(i) ResNet, (j)–(l) UNet, (m)–(o) FDUNet, (p)–(r) TNet, and (s)–(u) the proposed Dual SwinUNet models.

Figures 7(a)–7(c) illustrate phantoms containing 1, 3, and 5 graphite rods placed at different positions inside an agar phantom with intralipid added to act as scatterers. The corresponding backprojection reconstructed outputs are shown in Figs. 7(d)–7(f). The reconstructed images indicate that the graphite rods were partially recovered, but few artifacts exist in the image. Notably, in Fig. 7(e), one of the rods did not appear as a clear dot and instead generated a large circular artifact at the boundary, which could be misinterpreted as a false structure. In Fig. 7(f), the central rod was reconstructed correctly, whereas only four out of five rods were recovered because the fifth rod was positioned outside the illumination region and did not generate an acoustic signal. The remaining peripheral rods appeared distorted, i.e., reconstructed as arcs instead of dot/points. A possible reason for this could be sample breakage due to the fragile nature of the phantom. As the phantom experiences force toward the base of the water tank, this may have led to partial breakage or loose contact with the phantom holder boundary, causing movement of the phantom during data acquisition and resulting in the observed distortions. Contrast stretching was applied to the reconstructed images for further enhancement.

The enhanced reconstructed images were subsequently processed using several state-of-the-art deep learning networks, including ResNet, UNet, FDUNet, TNet, and the proposed Dual SwinUNet, with the corresponding outputs shown in Figs. 7(g)–7(u). ResNet failed to recover the background and, in some cases, did not reconstruct the structures accurately, primarily due to the absence of skip connections, as illustrated in Figs. 7(g)–7(i). The artifacts are indicated using red arrows in Figs. 7(g) and 7(i). UNet and TNet demonstrated improved suppression of artifacts surrounding the structures; however, both methods introduced noisy backgrounds, as shown in Figs. 7(j)–7(l) and 7(p)–7(r), respectively. Their overall performance was comparable; however, one of the three rods was not clearly distinguishable with UNet or TNet, as shown by red arrows in Figs. 7(k) and 7(q). FDUNet reduced artifacts while simultaneously suppressing background noise to some extent, which resulted in an improved image contrast, as highlighted in Figs. 7(m)–7(o). Finally, the proposed dual SwinUNet [Figs. 7(s)–7(u)] approach effectively suppressed artifacts and further enhanced the image contrast. As none of the models were explicitly trained to correct structural distortions, the overall shape remained consistent with that in the backprojected images. Nevertheless, the dual SwinUNet model achieved superior contrast enhancement and provided more accurate delineation of structure boundaries compared with the other methods.

We have evaluated the network performance using the gCNR as the quantitative metric for the phantom data, indicated in Fig. 7. The observations from gCNR values were consistent with the qualitative analysis. Importantly, the highest gCNR values were consistently obtained with the proposed dual SwinUNet, further confirming its effectiveness.

### 3.5 *In Vivo* Mice Imaging Data

**Fig. 8:**
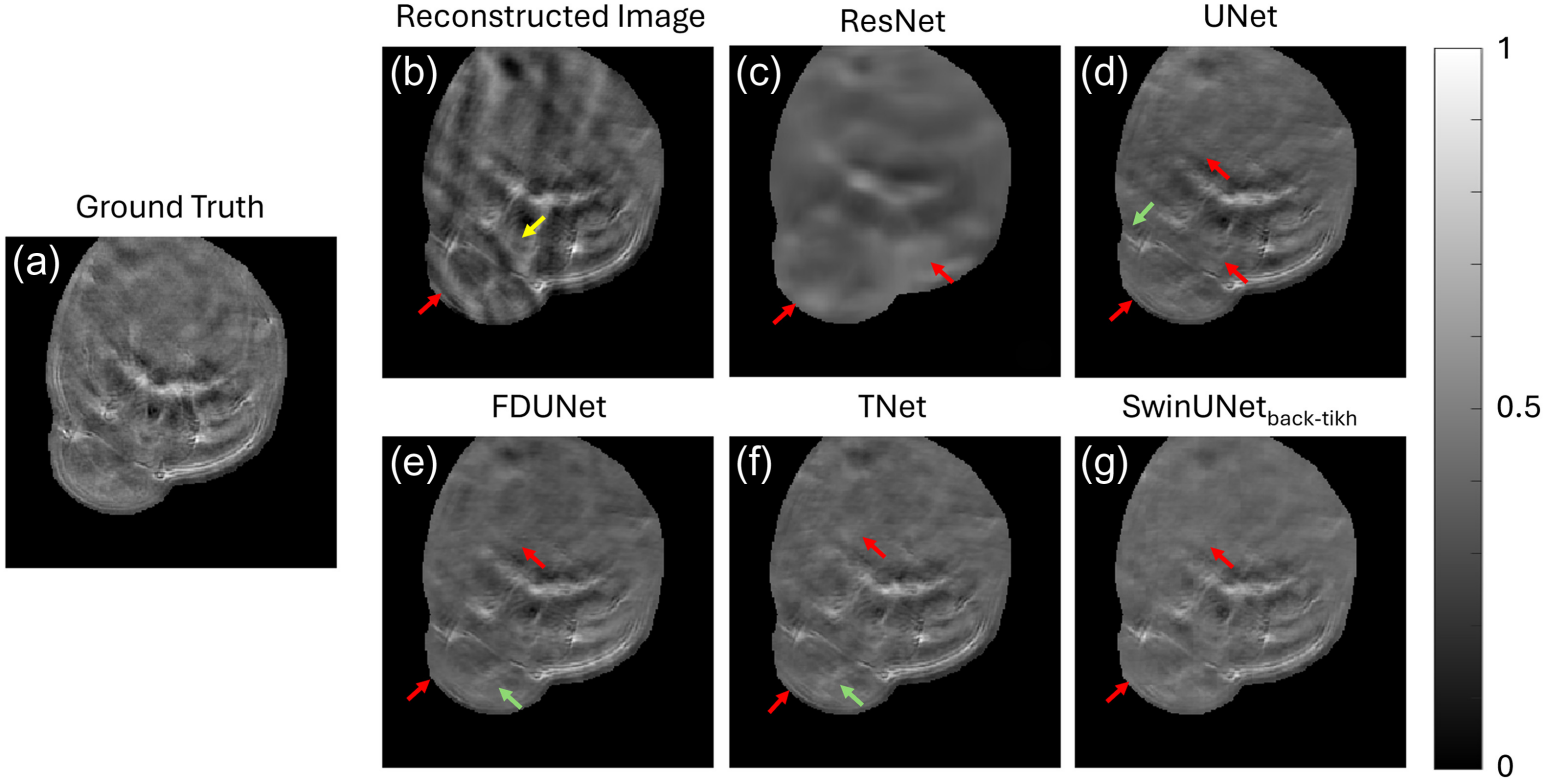
Comparison of the performance of backprojection reconstruction and various deep learning models using *in vivo* data. (a) Ground-truth image. (b) Backprojection reconstructed image. (c) Image reconstructed using ResNet. (d) Image reconstructed using UNet. (e) Image reconstructed using FDUNet. (f) Image reconstructed using TNet. (g) Image reconstructed using SWIN-UNet back-tikh. The green arrows indicate blurred structures, and the structures that have not been recovered and false structures are represented by red and yellow arrows, respectively.

The *in vivo* mice imaging data were collected with 256 transducers having an angular coverage of 270 deg. The images reconstructed using the full data were considered the ground truth, as shown in Fig. 8(a). To evaluate the limited-data setting, we have chosen the sinogram data from 100-transducer channel having an angular coverage of 135 deg. The reduced channel data were filtered, and image reconstruction using backprojection and Tikhonov regularization was performed using the filtered data. Figure 8(b) represents the backprojection reconstruction with limited data; as expected, few structural artifacts appeared in the reconstructed image; these artifacts manifested as false features (highlighted with yellow arrow). Note that a few structures were not accurately recovered (highlighted using red arrows in Fig. 8(b)). Figures 8(c)–8(g) represent the predicted outputs for different DL models, i.e., ResNet, UNet, FDUNet, TNet, and SwinUNet. Furthermore, ResNet has not recovered any of the structures [indicated using the red arrows in Fig. 8(c)]. The performances of UNet, FDUNet, and TNet were similar. The larger structures were recovered accurately; however, small features were missing (highlighted using a red arrow in Figs. 8(d)–8(f)]. The green arrow indicated the blurred features in Fig. 8(d)–(f). The proposed SwinUNet was found to recover sharp features in the tumor region, which appeared to be blurred in all the other networks’ output. There are still a few structures that were not clearly visible in Fig. 8(g) (highlighted with red arrows). We have evaluated the network performance using the quantitative metrics, i.e., SSIM and gCNR for the *in vivo* mice imaging data, as shown in Table 5. The quantitative results aligned well with Fig. 8. The SwinUNet achieved the highest SSIM for the *invivo* mice dataset, indicating superior structural similarity to the ground truth and better overall structure recovery than the other networks, whereas FDUNet attained slightly higher gCNR, and SwinUNet maintained competitive gCNR values that still reflected good foreground–background separation.

**Table 5:** Quantitative comparison (in terms of SSIM and gCNR) of backprojection and Tikhonov-regularized reconstructed image with 100 transducers and the other deep-learning-based network performance for *in vivo* mice imaging data.

Network	100 transducers	
	SSIM	gCNR
Backprojection reconstructed image	0.603±0.108	0.599±0.099
Tikhonov reconstructed image	0.636±0.125	0.632±0.059
ResNet	0.216±0.084	0.676±0.152
UNet	0.709±0.092	0.714±0.116
FDUNet	0.735±0.083	0.743±0.110
TNet	0.748±0.080	0.701±0.112
${\mathrm{Swin}\text{-}\mathrm{UNet}}_{\mathrm{back}\text{-}\mathrm{tikh}}$	0.783±0.069	0.721±0.085

Note: bold values indicate the best performance for each evaluation metric across all methods evaluated.

### 3.6 Ablation Study

**Fig. 9:**
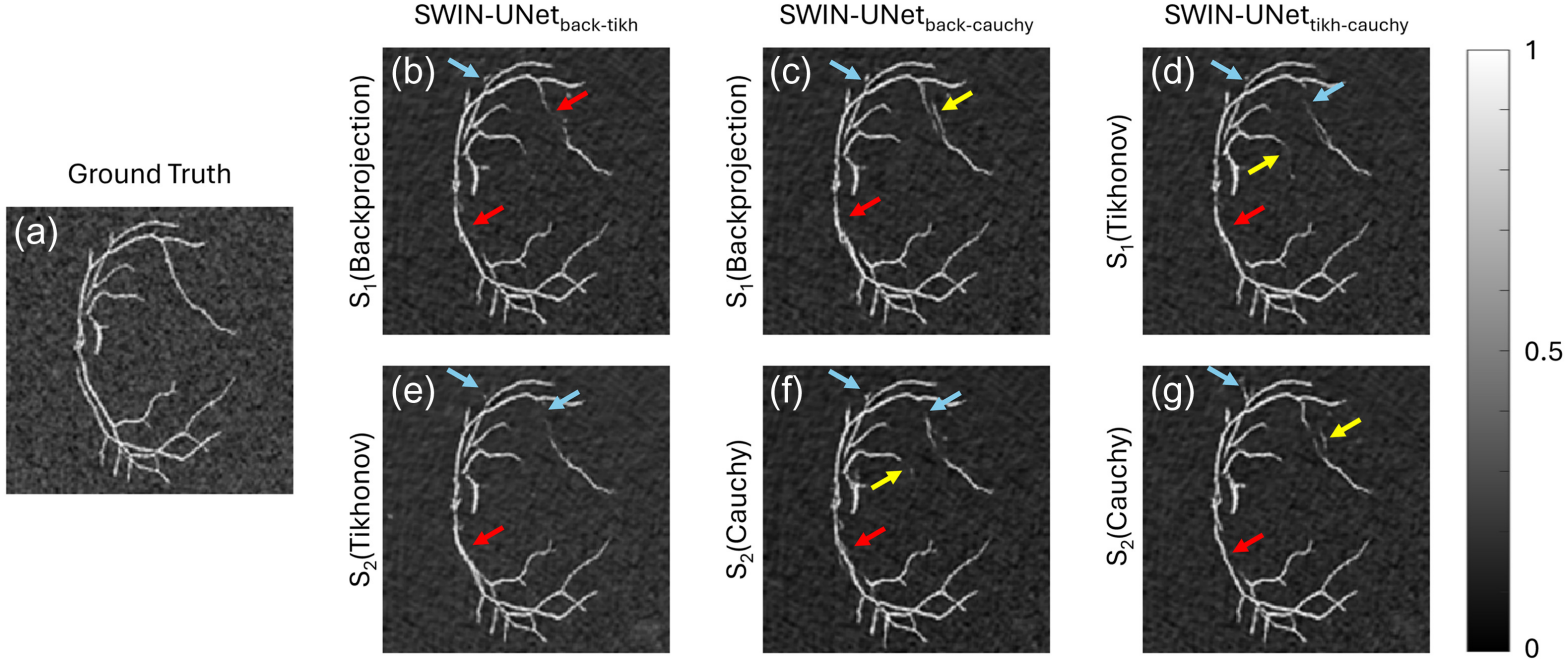
SWIN-UNet performance with different reconstructed image pairs as inputs for 20 dB SNR. (a) Ground truth. (b) SWIN-UNet output corresponding to backprojection reconstruction, trained with backprojection and Tikhonov reconstruction pairs. (c) SWIN-UNet output corresponding to backprojection reconstruction, trained with backprojection and Cauchy reconstruction pairs. (d) SWIN-UNet output corresponding to Tikhonov reconstruction, trained with Tikhonov and Cauchy reconstruction pairs, (e) SWIN-UNet output corresponding to Tikhonov reconstruction, trained with backprojection and Tikhonov reconstruction pairs. (f) SWIN-UNet output corresponding to Cauchy reconstruction, trained with backprojection and Cauchy reconstruction pairs. (g) SWIN-UNet output corresponding to Cauchy reconstruction, trained with Tikhonov and Cauchy reconstruction pairs. (a)–(g) Blue arrows represent the distorted/discontinuous and blurred structures, respectively; the structures that have not been recovered and false structures are represented by red and yellow arrows, respectively.

Figure 9(a) represents the ground truth image. For the ablation study, the SwinUNet was trained with three different pairs of input, i.e., backprojection–Tikhonov, backprojection–Cauchy, and Tikhonov–Cauchy-based reconstructed images. First, both the SwinUNet architectures ($S_{1}$ and $S_{2}$) of the dual SwinUNet model were trained using pairs of backprojection ($S_{1}$) and Tikhonov-regularized ($S_{2}$) reconstructed images as the input for the respective network. The trained networks, $S_{1}$ and $S_{2}$ were tested with backprojection ($S_{1}$) and Tikhonov-regularized ($S_{2}$) images respectively. The outputs of $S_{1}$ and $S_{2}$ networks are presented in Figs. 9(b) and 9(e) respectively, notably the performance of $S_{1}$ and $S_{2}$ were similar. Figure 9(b) and 9(e) had regions where the structures were missing (red arrows) and the distorted (blue arrows). Figure 9(c) and 9(f) represent the network outputs with backprojection ($S_{1}$) and Cauchy ($S_{2}$) based reconstructed images. Both $S_{1}$ and $S_{2}$ networks introduced false structures (yellow arrows) along with the distorted/discontinuous structure (as shown with blue arrows). Figures 9(d) and 9(g) are the outputs from the network trained with Tikhonov ($S_{1}$) and Cauchy ($S_{2}$)-based reconstructed images. The performance of Tikhonov–Cauchy pair is similar to the former two cases. All these cases suggest that dual SwinUNet with different pairs of reconstruction algorithms as input maintains consistent performance.

Furthermore, an ablation study was performed to compare the performance of single and dual SwinUNet architectures with different loss function variants. These observations are tabulated in Table 6. Note that the single SwinUNet model was trained with both backprojection and Tikhonov reconstruction outputs because the dual SwinUNet models require two inputs for training. Table 6 presents the quantitative metrics (SSIM, UIQI, and gCNR) for images reconstructed using backprojection and Tikhonov reconstruction methods. The performance of the single SwinUNet was evaluated using two loss functions: mean squared error (MSE) and noise-to-signal ratio (NSR), calculated between the predicted output and the ground truth. For the dual SwinUNet case, the performance was assessed using a combination of MSE and NSR. Table 6 indicates that the single SwinUNet architecture showed a slight improvement in SSIM, UIQI, and gCNR while using the MSE loss function compared with NSR loss. The dual SwinUNet architecture combined both the loss functions—wherein NSR was computed between the predicted output and the ground truth, and MSE was estimated between the predicted outputs of both networks. Table 6 illustrates that dual SwinUNet architecture performed the best compared with using standalone transformer type SwinUNet architecture. Therefore, adding the contrastive loss improves feature discrimination and structural consistency, whereas extending the framework from a single SwinUNet to the dual SwinUNet further enhances reconstruction quality by leveraging complementary information from two analytical reconstruction inputs.

**Table 6:** Single versus dual SwinUNet architecture comparison with various loss function combinations.

Loss function	Backprojection reconstruction			Tikhonov reconstruction		
	SSIM	UIQI	gCNR	SSIM	UIQI	gCNR
Reconstructed image	0.502±0.053	0.520±0.042	0.307±0.010	0.572±0.051	0.608±0.042	0.327±0.024
SwinUNet (NSR)	0.745±0.026	0.694±0.022	0.343±0.015	0.789±0.024	0.718±0.020	0.360±0.012
SwinUNet (MSE)	0.757±0.006	0.705±0.012	0.351±0.060	0.757±0.025	0.725±0.019	0.361±0.019
Dual SwinUNet (MSE + NSR)	0.856±0.029	0.769±0.027	0.373±0.011	0.895±0.024	0.803±0.020	0.390±0.023

Note: bold values indicate the best performance for each evaluation metric across all methods evaluated.

To further investigate the benefit of the proposed dual-network contrastive framework over the transformer-based backbone, we conducted an additional ablation in which the SwinUNet blocks in both branches were replaced by conventional convolutional architectures, specifically U-Net and FDUNet, while keeping the dual-network configuration, loss terms, and training protocol unchanged. Table 7 summarizes the resulting PSNR, SSIM, UIQI, and gCNR values for U-Net, FDUNet, and SwinUNet backbones under the same contrastive framework, showing that the dual-network contrastive setup improves reconstruction quality across all three backbones, whereas the SwinUNet-based model consistently achieves the highest scores in all metrics, indicating that the performance improvements arise from both the proposed dual contrastive formulation and the enhanced global feature modeling capability of SwinUNet.

**Table 7:** Quantitative comparison (using PSNR, SSIM, UIQI, and gCNR metrics) of different deep learning architectures in dual-network contrastive learning framework having a coverage of 135 deg.

Network	PSNR	SSIM	UIQI	gCNR
UNet	28.394±1.542	0.779±0.068	0.618±0.060	0.334±0.008
Dual UNet	32.172±1.983	0.827±0.011	0.704±0.012	0.344±0.056
FDUNet	30.916±1.346	0.795±0.067	0.645±0.054	0.336±0.006
Dual FDUNet	32.901±1.707	0.835±0.033	0.715±0.030	0.350±0.029
SwinUNet	30.159±2.173	0.757±0.006	0.705±0.012	0.351±0.060
Dual SwinUNet	34.261±0.012	0.856±0.029	0.769±0.027	0.373±0.011

In addition to CNN-based baselines, we further compared SwinUNet with two recent transformer-based U-Net variants, UNetR (ViT-based encoder), and MaxViT-UNet (multi-axis block and grid attention), trained under the same single-network reconstruction setting with identical data and loss configuration.

Table 8 shows that SwinUNet achieves slightly higher PSNR, SSIM, UIQI, and gCNR than both UNetR and MaxViT-UNet, indicating that it is a strong backbone choice for PAI reconstruction in our setting. Although SwinUNet’s window-based self-attention can in principle limit very long-range interactions in early layers, the shifted window strategy and hierarchical encoder–decoder design enable cross-window feature exchange and multiscale aggregation so that deeper layers can effectively capture global context; this is further complemented by the dual-network contrastive learning scheme, which enforces global consistency through the sinogram-domain forward model.

**Table 8:** Quantitative comparison (PSNR, SSIM, UIQI, and gCNR) of standalone transformer-based UNet variants (UNetR and MaxViT-UNet) and SwinUNet.

Network	PSNR	SSIM	UIQI	gCNR
UNetR	29.178±1.645	0.746±0.008	0.678±0.012	0.344±0.060
MaxVit-UNet	30.145±1.848	0.756±0.006	0.696±0.011	0.345±0.058
SwinUNet	30.159±2.173	0.757±0.006	0.705±0.012	0.351±0.060

Finally, we examined the effect of the Swin Transformer window size by comparing the default configuration with a window size 7 to a larger window size of 14 while keeping the dual-network contrastive framework, loss functions, and training protocol unchanged. As summarized in Table 9, the window size 7 model achieves higher PSNR, SSIM, UIQI, and gCNR than the window size 14 model, indicating that smaller windows provide more effective local modeling of fine vascular structures and subtle boundaries in PAI, whereas larger windows slightly increase the receptive field at the cost of oversmoothing and reduced sensitivity to fine details.

**Table 9:** Quantitative comparison (using PSNR, SSIM, UIQI, and gCNR metrics) of dual SwinUNet models with different window sizes.

	PSNR	SSIM	UIQI	gCNR
Window size=7	30.159±2.173	0.757±0.006	0.705±0.012	0.351±0.060
Window size=14	29.525±1.912	0.749±0.006	0.686±0.014	0.347±0.061

To complement the structural and perceptual metrics reported in this study, PSNR has also been included in the later experiments. The PSNR values follow the same overall trend as SSIM, UIQI, and gCNR, with SwinUNet consistently achieving superior reconstruction performance compared with the other models. This indicates that the proposed method improves not only visual quality and structural fidelity but also pixel-level reconstruction accuracy.

To investigate the effect of the loss weighting strategy, we performed an ablation study using four different settings:

• Reduced contrastive: reduced the contribution of contrastive losses: nsr1=1/4, nsr2=1/4, mse=1/4, contrastive neg=1/8, contrastive pos=1/8
• Reduced NSR: reduced the contribution of NSR losses: nsr1=1/8, nsr2=1/8, mse=1/4, contrastive neg=1/4, contrastive pos=1/4
• Reduced MSE: reduced the contribution of MSE loss: nsr1=2/9, nsr2=2/9, mse=1/9, contrastive neg=2/9, contrastive pos=2/9
• Equal weights: uniform/equal weighting (1/5 or 0.20 for all losses).

The quantitative results in Table 10 show that the equal-weight configuration yields the best overall performance in terms of PSNR, SSIM, UIQI, and gCNR. This suggests that assigning equal weights to the image-domain and sinogram-domain objectives provides a suitable trade-off for the proposed dual SwinUNet framework.

**Table 10:** Quantitative comparison (using PSNR, SSIM, UIQI, and gCNR metrics) of different weights to different loss functions.

	PSNR	SSIM	UIQI	gCNR
Reduced contrastive	33.124±2.517	0.844±0.006	0.749±0.008	0.365±0.061
Reduce NSR	33.151±2.434	0.845±0.006	0.750±0.008	0.366±0.061
Reduce MSE	33.710±2.316	0.846±0.005	0.752±0.007	0.365±0.059
Equal weights	34.261±0.012	0.856±0.029	0.769±0.027	0.373±0.011

To evaluate the trade-off between computational cost and reconstruction quality, we compared the single and dual SwinUNet configurations.

Table 11 shows that the dual-network design significantly improves PSNR, SSIM, UIQI, and gCNR relative to the single-network baseline. Importantly, because only one SwinUNet is used during inference, the proposed framework does not increase deployment latency, thereby preserving its suitability for near real-time photoacoustic imaging applications.

**Table 11:** Quantitative comparison (using PSNR, SSIM, UIQI, and gCNR metrics) of single versus dual SwinUNet.

	PSNR	SSIM	UIQI	gCNR
Single SwinUNet	30.159±2.173	0.757±0.006	0.705±0.012	0.351±0.060
Dual SwinUNet	34.261±0.012	0.856±0.029	0.769±0.027	0.373±0.011

## 4 Discussion and Conclusion

The input images to the network were generated using backprojection, Tikhonov regularization, and Cauchy-based penalization methods. The regularization parameters applied for the Tikhonov and Cauchy-based penalized reconstructions were ($1\times 10^{6}$) and ($4\times 10^{9}$), respectively. The proposed network architecture processes a pair of inputs, specifically two reconstructed images obtained via different reconstruction algorithms, each having different imaging characteristics. These inputs are transmitted to two separate SwinUNet networks, wherein each network is designed to extract features from the image domain. The predicted outputs generated by both networks, i.e., ($S_{1}$) and ($S_{2}$), are transformed into the sinogram domain using a forward model. The loss function is computed not only based on the predicted images but also on the sinograms of these outputs, ensuring that both domains contribute to improving the learning process. The network was trained using both positive and negative input pairs simultaneously. Positive pairs refer to inputs that share the same ground truth, whereas negative pairs correspond to inputs with different ground truths. This contrastive training approach enhances the network’s ability to differentiate between similar and dissimilar inputs, thereby improving overall reconstruction accuracy.

Contrastive learning has been effectively applied in various medical imaging tasks, including image segmentation tasks^58^ and image reconstruction for modalities such as magnetic resonance imaging (MRI) and computed tomography (CT).^59^ In image reconstruction, two primary strategies have emerged: (1) patch-based contrastive learning, where patches from the same image (from a single image reconstruction algorithm) are labeled as positive or negative based on spatial proximity or semantic similarity;^60^ and (2) cross-domain contrastive learning, which contrasts data from different modalities or domains to learn domain-invariant representations.^61^ However, contrastive learning has not been previously explored for solving image reconstruction problem in photoacoustic imaging. To the best of our knowledge, this is the first application of contrastive learning to solve photoacoustic image reconstruction. Our approach differs from prior strategies by operating entirely within a single modality and using full reconstructed images rather than patches. This formulation enables the model to learn reconstruction invariant features while preserving semantic consistency. More importantly, all the prior works have either used analytical or model-based reconstruction as input for deep learning models. To our knowledge, we have for the first time considered inputs from different reconstruction algorithms (having different characteristics) to improve the learning process with a transformer-like architecture, i.e., SwinUNet. The developed dual SwinUNet contrastive learning framework can be extended to other image reconstruction problems in MRI and CT.

The proposed network has been trained and tested under various conditions, including different numbers of transducers, varying angular coverage, and different noise levels. The dual SwinUNet architecture outperformed other state-of-the-art deep learning networks, such as ResNet, UNet, TNet, and FDUNet, under all of the above-mentioned conditions. The pre-trained network (with simulated vasculature data) was further retrained on the experimental *in vivo* mice data with the transducer configuration of 135 deg angular coverage and 100-transducer channels, for 40 epochs, and tested for the same. When the number of transducer channels was reduced from 100 to 40, a significant quantitative improvement was observed in SSIM (70% to 97%), UIQI (46% to 60%), and gCNR (23% to 90%) metrics with respect to the backprojection reconstructed image. Similarly, SSIM improved from 65% to 69%; UIQI improved from 40% to 46%; and gCNR improved from 6% to 23% (compared with backprojection reconstruction) when the angular coverage was reduced from 135 deg to 70 deg. Last, the SSIM, UIQI, and gCNR metrics improved from 70% to 83%, 46% to 50%, and 23% to 50%, respectively, when the data SNR was reduced from 40 to 20 dB.

The network was trained with various combinations of input pairs, including: (a) analytical–analytical reconstruction methods, where both inputs are reconstructed using analytical approaches, i.e., backprojection-time reversal; (b) analytical-model-based reconstruction methods, where one input is from an analytical method and the other from a model-based method; (c) model-based reconstruction methods, where both inputs are reconstructed using model-based approaches [see Fig. 9]. The performance across these combinations was found to be similar, indicating the robustness of the proposed network across different reconstruction methods. Therefore, the specific input combination used in each figure or table was chosen only for presentation purposes, whereas the overall methodology remains consistent across experiments. Despite the different combinations, the proposed dual SwinUNet architecture consistently outperforms other deep learning networks in terms of accuracy and computational efficiency. This demonstrates the network’s capability to generalize well across various reconstruction algorithms and its superior performance in photoacoustic imaging (PAI) reconstruction.

To better quantify the relative contributions of the proposed dual-network contrastive framework and the choice of backbone, we fixed the dual contrastive setup and systematically varied the backbone architecture, considering U-Net, FDUNet, and SwinUNet within the same training protocol. In a complementary experiment, we compared SwinUNet with two recent transformer U-Net variants, UNETR, and MaxViT-UNet (Table 8) and observed that all transformer backbones outperform conventional CNNs, with SwinUNet providing superior or comparable performance across PSNR, SSIM, UIQI, and gCNR. These findings, together with the ability of transformers to model long-range dependencies, produce semantically structured feature embeddings, and aggregate multiscale information, suggest that transformer backbones are particularly well suited for contrastive learning in PAI and justify the choice of SwinUNet as the backbone for the proposed dual-network framework.

Table 12 compares the number of parameters across five different networks: ResNet, UNet, FDUNet, TNet, and SwinUNet. ResNet has the fewest parameters, whereas the other networks have significantly larger parameter counts. UNet and TNet have around 31 million parameters, and FDUNet has the highest number of parameters. Meanwhile, SwinUNet has a relatively lower number of parameters compared with the other deep-learning-based architectures, i.e., 27 million parameters. These networks were also compared based on their forward/backward pass size and total network size. ResNet has the smallest forward/backward pass size of 210.64 MB and a parameter size of just 1.54 MB, resulting in a total size of 212.37 MB. By contrast, UNet, FDUNet, and TNet each have similar forward/backward pass sizes of around 780 MB. Their total sizes ranged from 899.52 to 2529.49 MB. Notably, SwinUNet has a forward/backward pass size of 502.30 MB, which is considerably lower than that of the FDUNet architecture, and a parameter size of 103.55 MB. As a result, SwinUNet’s total size is 606.05 MB, making it significantly smaller than the other networks. This reduced total size further emphasizes the advantages of SwinUNet, particularly in terms of lower memory and computational requirements, which can lead to faster processing and less resource consumption, making it an ideal choice for practical applications where efficiency is key.

**Table 12:** Comparison of the number of parameters, parameter size, network size, training, and inference time for the different DL models used in this work.

Model	Number of params	Forward/backward pass size (MB)	Params size (MB)	Total size (MB)	Training time (sec/epoch)	Inference time (ms/image)
ResNet	403,289	210.64	1.54	212.37	3.2	8.5
UNet	31,036,481	780.94	118.39	899.52	8.7	15.3
FDUNet	31,964,929	2406.36	121.94	2528.49	10.4	21.8
TNet	31,036,481	780.94	118.39	899.52	9.8	16.1
Swin-UNet	27,145,824	502.30	103.55	606.05	11.7	18.6

In this work, the acoustic forward operator used to map the SwinUNet outputs back to the sinogram domain assumes a homogeneous speed-of-sound distribution. This assumption is consistent with the numerical data generation and the reconstruction settings used in our experiments. However, the real tissue often exhibits spatially varying speed of sound, which can introduce phase aberrations and reduce reconstruction accuracy. The proposed dual SwinUNet framework is not inherently limited to a constant speed-of-sound model, instead it requires only a forward operator that maps the reconstructed image into the corresponding measurement domain. Therefore, the current formulation can be extended to two- or multicompartment speed-of-sound models in future studies without changing the network architecture.

The proposed model was evaluated across multiple noise levels, detector counts, and angular coverages in simulation and was then transferred to two distinct experimental settings, namely, agar phantoms with point absorbers and *in vivo* mouse imaging. In both cases, the proposed framework improved contrast and structural fidelity over the CNN-based baselines, suggesting that the learned representation is not limited to a single acquisition condition. These observations indicate that the dual SwinUNet can generalize effectively across both synthetic and experimental PAT settings, despite differences in measurement geometry and data characteristics.

Although the proposed network demonstrates state-of-the-art performance across the tested photoacoustic reconstruction scenarios, it may produce minor artifacts or reduced structural fidelity when the input data are affected by extreme noise levels, conditions outside the training distribution, or acquisition-specific artifacts. In such cases, the network can occasionally confuse weak noise patterns with true anatomical structures, leading to smoothing. In addition, the present formulation does not explicitly model spatial variations in acoustic speed, which may further limit reconstruction sharpness in heterogeneous tissue. These observations indicate that, although the dual SwinUNet is robust in the evaluated settings, its performance can still degrade under strongly mismatched imaging conditions.

The current work has used a transformer-based architecture as a backbone (for dual-network- and contrastive learning-based models), which has great potential for pre-clinical and clinical photoacoustic imaging. However, future research can explore the utility of lightweight models to reduce the training time. The major focus of this work is on deterministic, physics-driven reconstruction networks with explicit data-fidelity terms, whereas diffusion- and GAN-based approaches, which often act as image-domain generative priors or post-processors, can be considered promising future extensions of the proposed dual-network contrastive framework.

Although this study focuses on photoacoustic tomography (PAT), the proposed dual SwinUNet framework, with its hierarchical attention and contrastive representation learning, holds promise for broader application across other photoacoustic modalities such as photoacoustic microscopy,^62^ photoacoustic mesoscopy,^63^ functional photoacoustic imaging,^64^ and multispectral photoacoustic imaging.^65^ These modalities differ in resolution, depth, and contrast mechanisms, but share similar reconstruction challenges that could benefit from our model’s ability to learn robust spatial features and discriminate subtle structural variations.

## Data Availability

Data underlying the results presented in this paper are not publicly available at this time but may be obtained from the authors upon reasonable request.
